# Realization of Intermolecular Interactions as a Basis for Controlling Pervaporation Properties of Membranes Made of Aromatic Polyamide-Imides

**DOI:** 10.3390/membranes15010023

**Published:** 2025-01-13

**Authors:** Svetlana V. Kononova, Galina N. Gubanova, Galina K. Lebedeva, Elena V. Kruchinina, Elena N. Vlasova, Elena N. Popova, Natalya V. Zakharova, Milana E. Vylegzhanina, Elena A. Novozhilova, Ksenia V. Danilova

**Affiliations:** 1 Branch of Petersburg Nuclear Physics Institute Named by B.P. Konstantinov of National Research Centre «Kurchatov Institute»—Institute of Macromolecular Compounds, Bolshoy pr. 31, 199004 Saint Petersburg, Russia; constanta2011.lebedeva@mail.ru (G.K.L.); popovaen@hq.macro.ru (E.N.P.); na_zar@inbox.ru (N.V.Z.); v.e.milana@gmail.com (M.E.V.); 2 Saint-Petersburg State Institute of Technology, Technical University, 190013 Saint Petersburg, Russia

**Keywords:** polyamide-imides, hydrophilic polymers, polycondensation, structure, intermolecular interactions, thermogravimetric analysis, pervaporation

## Abstract

New aromatic co-polyamide-imides (coPAIs) containing both carboxyl and hydroxyl groups in the repeating units were synthesized for the first time. Transport, thermal and morphological properties of dense nonporous membranes from PAIs obtained using the diacid chloride of 2-(4-carboxyphenyl)-1,3-dioxoisoindoline-5-carboxylic acid and diamines 5,5′-methylene-bis (2-aminophenol)) and 3,5-Diaminobenzoic acid, taken in molar ratios of 7:3, 1:1, and 3:7, have been studied. High levels of membrane permeability accompanied by high selectivity for mixtures of liquids with significantly different polarities were determined by realization of intra- and intermolecular interactions in polymer, which was proved by thermal analyses and hydrodynamic characteristics of coPAIs. This effect is discussed in the context of the effectiveness of intermolecular interactions between polymer chains containing carboxyl and hydroxyl functional groups.

## 1. Introduction

Pervaporation technologies are now widely applied in the areas of the world industry employing hybrid schemes of separation of liquid substances. However, the efficiency of these technologies depends on characteristics of the used membranes; thus, the development of membranes with desired properties for specific applications remains an urgent research task [1,2,3,4,5,6].

Pervaporation membranes should be effective in the processes of separation by the diffusion–sorption mechanism combined with other mechanisms of selective transport of liquids (such as the mechanism of facilitated transport of membrane-penetrating substances (penetrants)). Consequently, the most interesting approaches involve the introduction of active functional groups (intended for various applications) into membrane-forming polymers. By changing reactivity of these groups through chemical modification of polymers and varying the conditions of membrane preparation or post treatment, it is possible to influence (ideally, to regulate) activity of functional groups in transport processes and simultaneously control physico-chemical characteristics of the corresponding membrane material [7,8]. Interesting but ambiguous effects of this type were revealed after the introduction of hydroxyl and carboxyl groups into the polymer matrix of composite materials (so that the resulting composites consisted of a combination of hydroxyl- and carboxyl-containing macromolecules). Such composite materials were prepared in the form of polymer blends and interpolymer complexes, including polyelectrolyte complexes [9,10,11].

The introduction of hydrophilic functional groups into polymer chains was aimed at controlling pervaporation separation of water–organic mixtures. However, in the cases of selective transport of aliphatic alcohol–water mixtures through membranes made of hydroxyl- or carboxyl-containing polymers, the separation process was not unambiguous, and the result did not depend only on the amount of the mentioned functional groups in macromolecules. The steric factor (i.e., the nature of formed transport channels responsible for membrane permeability), which influences the degree of involvement of functional groups in the process of mass transfer of penetrants, played an important role in separation [12,13].

Of particular interest are cross-linked polymer systems based on hydroxyl- and/or carboxyl-containing polymers, in which cross-linking occurs at the expense of hydrogen bonding (physical cross-linking) or as a result of chemical reactions with the formation of new chemical compounds (chemical cross-linking) [14,15,16,17,18]. The formation of these cross-links provides an additional opportunity to regulate the number of functional groups participating in the transport of penetrant molecules (i.e., the active functional groups) and to influence the packing density of polymer chains. Thus, it will be possible to control both sorption and diffusion factors of separation of liquid mixtures.

Recall that the polymeric membranes discussed above have not yet been thoroughly studied, and the search for optimal conditions for their production and application remains a promising topic. However, a logical question (and with it the need for new research) has arisen as to how the simultaneous introduction of hydroxyl- and carboxyl-containing fragments into the macromolecule of a membrane-forming polymer will affect the properties of pervaporation membranes. The literature contains only a few works devoted to pervaporation with “bifunctional” polymers (mostly polysaccharides) [19]. Our previous studies have demonstrated that the synthesis of polycondensation polymers containing both carboxyl and hydroxyl functional groups in each macromolecule is a promising approach to the production of pervaporation membranes. This approach allows one to realize intra- and intermolecular interactions involving functional groups.

Aromatic polyamide-imides were chosen as the most interesting objects for this study; our previous work was devoted to composite pervaporation membranes based on these polymers. The investigation of (co)polymers with weakly dissociating acid groups has been carried out; the objects of this earlier work were (co)polyamide-imides with rigid links containing diaminobenzoic acid fragments [12]. It has been shown that when loading diamines (diaminodiphenyl ether and diaminobenzoic acid) into the reaction mixture in the 3:7 ratio, the extended blocks containing carboxyl groups were found in the synthesized polymer, and conversely, when the comonomer ratio was 7:3, hydrophobic blocks were present in the resulting copolymer.

Transport properties of the obtained polymers correlated well with the results concerning the membrane surface characteristics, their domain structure, and the presence of strongly bound residual solvent.

We have attempted to control the hydrophilic–lipophilic balance of the polymer membrane by introducing hydroxyl-containing fragments into the polyamide-imide chain [13]. In the present work, a new approach to the modification of polyamide-imides was used: the fragments (units) containing hydrophilic (hydroxyl) groups were introduced at the ortho-position to the amide bond. The presence of hydroxyl groups in diamine at the ortho-position to the amino groups (the fragments participating in polycondensation) allowed us to carry out further transformations in the polymer chain: dehydrocyclization with the formation of hydrophobic fragments in the polymer. These reactions were supposed to regulate not only the level of hydrophilicity of the obtained polymers, but also the set of their useful properties, including mechanical and transport characteristics.

It should be noted that the presence of a small amount of film-forming solvent, detected by various methods, contributed to the formation of the membrane film, which has been observed earlier in the studies on the formation of polyamide-imide diffusion membranes [20]. Presumably, the stability of this type of membranes is related to the presence of physically bound residual solvent (N-methyl-2-pyrrolidone) in the polymer films; effective release of this solvent is detected only at temperatures above 200 °C.

The analysis of the presented results allowed us to conclude that further studies are necessary to thoroughly investigate the structure of the films obtained from the synthesized polymers at various stages of their formation, taking into account structural changes occurring during solvent evaporation and then as a result of pervaporation.

It remains unclear whether the assumed competing processes of formation and breaking of hydrogen bonds involving hydroxyl groups are realized and how they affect physico-chemical characteristics and film-forming properties of the studied polymers. It is most interesting to investigate the process of formation of a membrane supramolecular structure since the synthesized copolymers contain extended fragments of macrochains that differ significantly in the amount of hydroxyl functional groups (i.e., have block structure). Then, it is necessary to understand in which way the formed structure influences the ability of membranes to perform mass transfer of liquids (penetrants) of different polarities. First of all, this applies to liquids capable of physical interaction with hydroxyl groups of polymers with the formation of hydrogen bonds.

In the present study, we attempted to synthesize new aromatic (co)polyamide-imides containing both carboxyl and hydroxyl groups in the repeating units. There are no scientific works in the literature that analyzed polymers containing the above-mentioned groups simultaneously, and in different ratios, as well as non-porous films based on them for pervaporation processes. The literature describes polymers with simultaneous content of different functional groups in the chain, and films based on them for gas separation processes, selectivity, permeability, and thermal stability have been studied [21,22,23,24]. In particular, the introduction of various bulky pendant groups into the polymer precursor, for example, 4-tert-butylbenzoyl or p-toluene, is discussed, leading to an increase in the selectivity and permeability of the polymer membrane [23].

However, we are interested in the functionalization of polymers, which can lead to competitive intra- and intermolecular interactions. The implementation of interactions involving hydroxyl and carboxyl groups can lead to a change in the hydrophilic, hydrophobic, and other properties of polymers associated with the number of free functional groups, structural features and thermophysical characteristics of film membranes. Another goal was to investigate the influence of copolymer structural features on the complex of properties required for the formation of high-performance pervaporation membranes and to compare them with the corresponding homopolymers synthesized under similar conditions and containing only one type of functional groups (carboxyl or hydroxyl).

## 2. Experimental

### 2.1. Materials

3,5-Diaminobenzoic acid (DABA) (CAS: 535-87-5) and propylene oxide (Sigma Aldrich Merck KGaA, Darmstadt, Germany, 99%, CAS 75-56-9) were used without additional purification.

Diacid chloride of (2-(4-carboxyphenyl)-1,3-dioxoisoindoline-5-carboxylic acid) (diacid chloride of CPDCA) was prepared by condensing trimellitic acid anhydride and *p*-aminobenzoic acid followed by transformation of CPDCA into diacid chloride by the reaction between CPDCA and thionyl chloride according to the procedure described in [25]. 5,5′-Methylenebis(2-aminophenol) (DADHyDPhM) was prepared according to the technique described in [26] by condensation of 2-aminophenol with formaldehyde in the presence of sulfuric acid and sodium sulfite.

### 2.2. Preparation of Polymers

**Figure 1:**
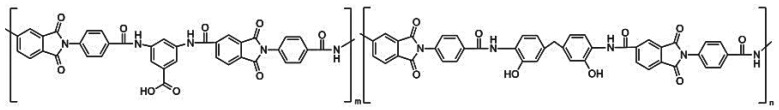
Chemical structure of polyamide-imides: m = 1, n = 0 (PAI I); m = 0, n = 1 (PAI II); m = 0.7, n = 0.3 (coPAI III); m = n = 0.5 (coPAI IV); m = 0.3, n = 0.7 (coPAI V).

Synthesis of PAI I (Figure 1) was performed according to the following technique. 3,5-Diaminobenzoic acid (DABA, 0.76 g) was dissolved in 15 mL of N-methyl-2-pyrrolidone (NMP) containing no more than 0.035 wt.% of moisture at room temperature. Diacid chloride of 2-(4-carboxyphenyl)-1,3-dioxoisoindoline-5-carboxylic acid (diacid chloride of CPDCA, 1.79 g) was added to cooled solution at stirring, and the reaction mixture was left to stand at room temperature for 1.5 h. Then propylene oxide (0.7 mL) was added; the resulting mixture was stirred for 60 min.

Homopolymer PAI II and copolymers coPAI (III–V) were prepared in a similar way by the reaction between diacid chloride of CPDCA and DADHyDPhM or a mixture of diamines with various molar ratios between components (DABA: DADHyDPhM = 7:3 for coPAI III, 1:1 for coPAI IV, and 3:7 for coPAI V).

The values of reduced viscosity of 0.5 wt.% polymer solutions in NMP (*η*, dL/g) were the following: 1.07 (PAI I), 0.86 (PAI II), 1.00 (coPAI III), 0.77 (coPAI IV), 0.88 (coPAI V).

### 2.3. Preparation of Samples for Studies

Powder-like polymer samples were prepared by precipitation into ethanol from the initial solution.

Dense PAI (I–V) polymer films were prepared by casting 15 wt.% solutions of polymers in NMP onto a glass plate followed by stepwise drying at 50–100 °C in order to remove the solvent. The obtained films were separated from the glass plate, subjected to thermal treatment at 150 °C and then heated stepwise up to 300 °C (with a step of 50 °C).

### 2.4. Instruments and Methods

***IR spectra*** of homopolymer films containing diamines (DABA or DADHyDPhM) and copolymers prepared using diamine mixtures DABA: DADHyDPhM (molar ratios 7:3, 1:1, 3:7) were registered with the use of a Vertex 70 IR Fourier spectrometer (“Bruker” (Billerica, MA, USA)) at room temperature in the wavelength range from 400 to 4000 cm^−1^. The spectrometer was equipped with a ZnSe attenuated total reflectance (ATR) micro attachment (Pike). During registration of ATR spectra, the correction for light penetration depth depending on wavelength was made.

***^13^C NMR spectra*** were obtained using a Bruker AC-400 spectrometer (Billerica, MA, USA) in deuterated dimethylsulfoxide (DMSO–d_6_). The values of chemical shifts (δ) are given downfield from TMS as an internal standard.

***Kinematic viscosities*** of 0.5 wt.% solutions of the polymers in NMP were measured with the use of a VPZh-1 glass capillary viscometer.

***Heat resistance parameters*** of powder-like samples were investigated by thermogravimetric analysis (TGA) and differential thermal analysis (DTA) using a DTG-60 synchronous thermal analyzer (Shimadzu, Kyoto, Japan). Powdered sample (~5 mg) was placed into an open Al_2_O_3_ crucible and heated in air at a heating rate of 5 grad/min. Mass losses (m/m_o_) and temperature effects ΔT (heat absorption or release) were registered.

The results were used to determine heat resistance parameters of the studied materials, i.e., the temperatures at which polymer mass decreased as a result of thermodestruction by 5, 10, and 50%, respectively.

#### 2.4.1. Atomic Force Microscopy (AFM)

Atomic force microscopy studies were carried out using a Nanotop NT-206 atomic force microscope (ODO “Microtestmachines”, Gomel, Belarus) in the tapping mode under atmospheric conditions (NSC11/AlBS silicon cantilevers, the force constant: 1.5–5 N/m, the tip curvature radius: 10 nm). The experimental data were processed using the Surface Explorer 1.3.1.1 program.

#### 2.4.2. Differential Scanning Calorimetry (DSC)

Differential scanning calorimetry (DSC) analysis of the PAI and coPAI films was carried out using a Netzsch DSC 204 F1 heat flow differential scanning calorimeter (Netzsch, Selb, Germany) in the temperature range from 0 to 400 °C in argon atmosphere (argon flow rate: 25 mL·min^−1^, heating rate: 10 deg·min^−1^).

Thermogravimetric analysis of films was performed using a Netzsch TG 209 setup (Netzsch, Selb, Germany) in the temperature range from 30 to 600 °C at a constant heating rate in argon medium. The weight of tested samples was 2–3 mg.

#### 2.4.3. Determination of Molecular Weights and Hydrodynamic Characteristics of Polymers

Molecular weights *M_w_* of the PAI (I,II) and coPAI (III–V) samples, hydrodynamic radii *R*_h-D_ of macromolecules, and second virial coefficients *A*_2_ were measured by static and dynamic light scattering in dilute solutions in NMP and in a mixture of solvents (NMP + three drops of methanol). Light scattering was studied with a Photocor Complex setup (Photocor Instruments Inc., Moscow, Russia), the light source was a Photocor-DL diode laser (power: 5–30 mW, wavelength λ = 659.1 nm). The calibration of the instrument, i.e., determination of the instrument factor, was carried out using toluene (RV = 1.38 × 10^−5^ cm^−1^). The measurements were performed at scattering angles *θ* ranging from 45 to 135°. The correlation function of the scattered light intensity was recorded using a PhotocorPC2 correlator with 288 channels and processed using DynalS software (version 8.2.3, SoftScientific, Tirat Carmel, Israel). The experiments were performed at 21.0 °C.

Refractive index increments dn/dc were measured using an RA-620 refractometer (λ = 589.3 nm) (KEM, Kyoto, Japan).

Characteristic viscosity [*η*] was measured at 21 °C in NMP and in NMP–methanol mixture (NMP + 3 drops of methanol) using a Cannon-Manning-type Ostwald capillary viscometer (Cannon Instrument Company Inc., State College, PA, USA).

To evaluate the adhesion characteristics of the surfaces (wettability, surface energy), contact angles of different liquids for these surfaces were measured in the range from 1 to 180° with an accuracy of 0.1° using a DSA14 instrument (KRȔSS (Hamburg, Germany)). The range of surface tension measurements was 0.1–1000 mN/m, with an accuracy of 0.1 mN/m. Deionized water (with predominance of polar component of surface tension) and glycerol (with equal polar and dispersive components) served as test liquids.

Total surface energy and polar and dispersion components were calculated using the Fawkes method based on the measured contact angles of the two test liquids [27].

#### 2.4.4. Pervaporation Experiments

Pervaporation properties of the obtained dense membranes (non-porous films) were tested in the experiments involving different penetrants (water, ethanol, methanol, cyclohexane) using a non-continuous flow laboratory cell, as described in [20] with an operating membrane area of 1.38 × 10^−3^ m^2^ at a constant temperature of 40 °C. Permeate vapors were condensed using liquid nitrogen. The received permeate was weighed, and the flux value J [kg × m^−2^ × h^−1^] was estimated using the following equation:

$$J=m\times S^{-2}\times t^{-1}$$

where m is the mass (kg) of the penetrant permeated through membrane area S (m^2^) in a period t (h); P = J × *l* [µm × kg × m^−2^ × h^−1^] (permeation rate) is the flux of a penetrant normalized to the membrane thickness of 1 µm.

## 3. Results and Discussion

### 3.1. Preparation and Characterization of Polyamide-Imides

The first stage of the present research work involved the synthesis of polyamide-imides, which are the products of polycondensation of aromatic diamines with aromatic dibasic acid dichlorides containing imide rings.

**Figure 2:**
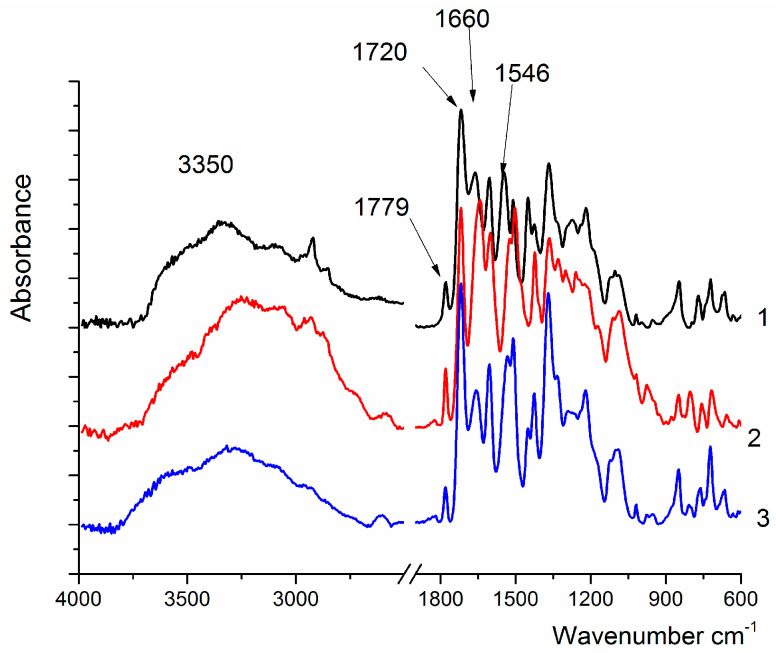
IR spectra of PAI I (1), PAI II (2), and coPAI IV (3).

The IR spectra of the obtained samples contain the absorption bands characteristic of all PAI (Figure 2). The peaks located near 1780 cm^−1^, 1720 cm^−1^, and 1369 cm^−1^ are assigned to imide rings, and another group of absorption bands are related to the amide group, namely: the bands in the region of 3250–3330 cm^−1^ (NH valence vibrations), 1644 cm^−1^ (C=O absorption), and 1524 cm^−1^ (NH deformation vibrations).

In the spectrum of coPAI IV, the broad band at 3600–3200 cm^−1^ (assigned to the vibrations of OH-groups) shifts toward 3700 cm^−1^; this can be attributed to the appearance of hydrogen bonds, which are weaker than those formed in homopolymers (Figure 1).

Chemical structure of the obtained polyamide-imides was also confirmed by ^13^C NMR spectroscopy. The results are presented in the Supplementary Materials.

In the ^13^C NMR spectra (DMSO-d_6_) of PAI I and coPAI (III–V) (the polymers with side carboxyl groups), a peak at δ = 174.2 ppm characteristic of the carbon bound to carboxyl group (HOO–CH=) was observed (Figure S1, Supplementary Materials).

The spectra of all polymers contain signals in the region of 122.9–135.0 ppm, characteristic of aromatic carbon atoms (–CH=) of benzene rings of the amine component and benzoimide rings of the acid (chloroanhydride) component (Figure S2, Supplementary Materials). The signals characteristic of the aromatic carbon atom bound to the nitrogen atom of the amine component (–HN–CN=) are localized in the 122.9–124.2 ppm region (Figure S2, Supplementary Materials).

In the spectra of all polymers, the signals characteristic of the quaternary carbon atom of the carbonyl group (>C=O) are observed in the 164.2–167.5 ppm range: the peaks of amide bond in the form of singlets (PAI I, PAI II) and duplets (copolymers), and the peaks of imide ring in the form of duplets (Figure S3, Supplementary Materials). The spectra of polymers with the hydroxyl group (PAI II and coPAI (III–V)) include the signals characteristic of carbon of benzene ring bound to the hydroxyl group (HO–CH=) in the range of 150–150.8 ppm (Figure S4) and the peak at 116.8 ppm characteristic of aromatic carbon atoms bound to the hydroxyl group (–CH=C<OH) (Figure S5, Supplementary Materials).

### 3.2. Pervaporation Properties of Membranes

The synthesized polymers were used to prepare continuous non-porous films (membranes) by solution casting as described in Section 2.

**Figure 3:**
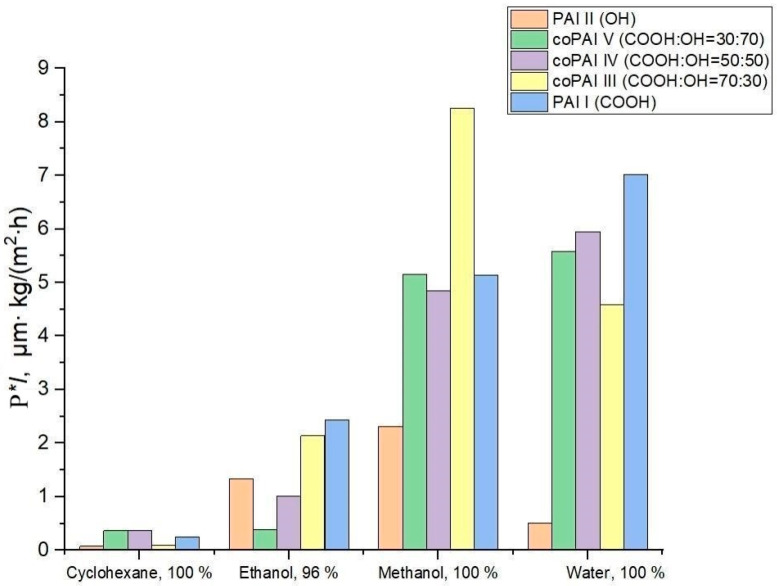
Values of flux normalized by thickness for different penetrants and membranes; the penetrants were tested in the order of increasing their polarity.

**Figure 4:**
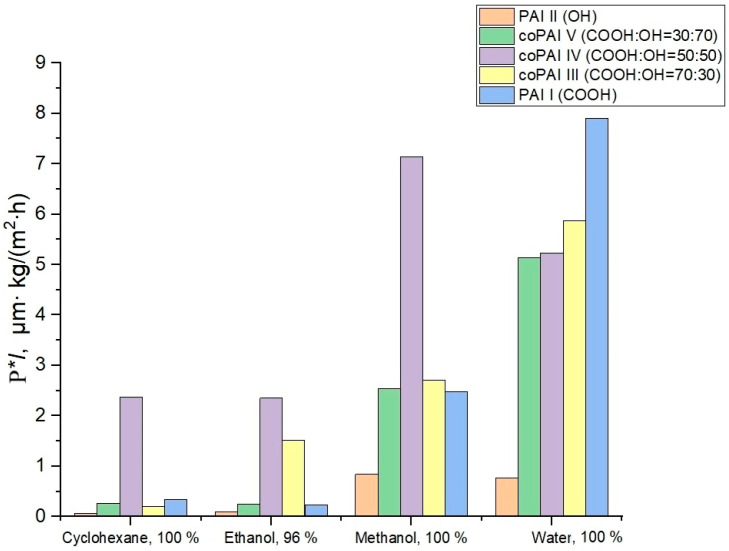
Values of flux normalized by thickness for different penetrants and membranes; the penetrants were tested in the order of decreasing their polarity.

Table 1, Figure 3 and Figure 4 give transport properties of the obtained membranes measured under pervaporation conditions with freezing at 40 °C during sequential permeation of penetrants of the cyclohexane–ethanol–methanol–water series (increasing polarity of a penetrant) and then in the reverse sequence (water–methanol–ethanol–cyclohexane, decreasing polarity of a penetrant).

The data given in Table 1 show that the PAI I membrane is more permeable than PAI II and (based on their water permeability) more hydrophilic. The higher the solvent polarity, the greater the rate of mass transfer of this solvent through PAI I. The same trend is observed in the case of PAI II, but the low water flux values of films made of this polymer are surprising.

The observed effect may be attributed to the formation of intramolecular hydrogen bonds, the presence of which is discussed in detail in our previous publication [13]. As a result, the majority of hydroxyl groups capable of interacting with water (hydration) are not reactive and thus are not capable of interactions. Interestingly, all the polymers considered have the highest alcohol permeability and, in particular, methanol permeability. The obtained results suggest that, unlike water transport (which depends on the level of hydrophilicity of a polymer determined by the amount of active –COOH and –OH functional groups), the transport of aliphatic alcohols is associated with other interactions between polymers and penetrant molecules. This conclusion should be supported by additional further specialized studies of the transport of aliphatic alcohols through polymers of the aromatic polyamide-imide class.

The change in cyclohexane permeability in the series of polymers 1 → 5, arranged according to the increase in the number of carboxyl groups in the macromolecules, can be related to the increase in the free volume in this series of polymers and is determined by the characteristics of the supramolecular structure. In this connection, we paid special attention to the investigation of structural and morphological features of the prepared polymers and possible changes in their structure depending on solvents (used both in the process of membrane formation and in the process of pervaporation).

The study of supramolecular structure of PAIs in membranes is all the more important because of the possible competition between intra- and intermolecular interactions in polymers between carboxyl and hydroxyl groups. The formation of membranes proceeds during solvent removal from molding solutions at heating (150 °C). In this process, the formation of ester bonds is possible. However, the extent to which this possibility is realized in practice and how this process affects the membrane structure and its stability was to be determined by additional studies; the results of these studies are presented below.

### 3.3. Thermal Analysis of Homopolymers and Copolymers

As was discussed in the previous section, analysis of the pervaporation properties of the investigated film membranes shows that their permeability is not directly related to the amount of hydrophilic carboxyl groups in the macromolecules. This may be observed if carboxyl groups in some macromolecules are not functionally active. This deactivation, in turn, may be caused by specific features of the supramolecular structure of the polymers. It is possible that some of the carboxyl groups are not reactive because they have already interacted with other reactive groups of the polymer or solvent (formation of hydrogen, ester, or other types of bonds). In connection with the above, it was reasonable to analyze thermophysical properties of the studied polyamide-imides, considering the presence of residual solvents in the films, as well as the possibility of structural transformations in polymers under conditions of membrane formation.

In order to establish thermophysical characteristics of coPAI films containing hydroxyl and carboxyl groups, samples heated at 150 °C were investigated by thermogravimetric analysis (TGA) and differential scanning calorimetry (DSC) (a series of pervaporation tests were carried out for these membrane samples).

**Figure 5:**
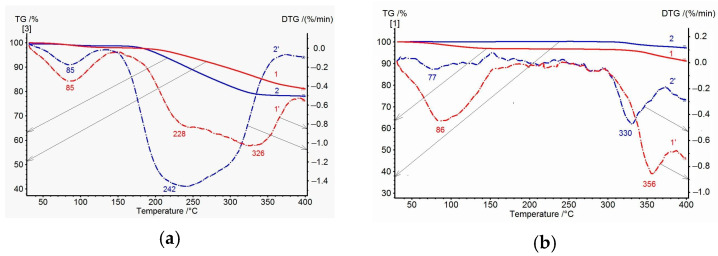
TGA (solid) and DTG (dotted) curves of homopolymer samples heated at 150 °C (**a**) and 300 °C (**b**): 1, 1′—PAI I and 2, 2′—PAI II.

Figure 5a shows the TGA and DTG data for PAI I (COOH) and PAI II (OH) homopolymer samples. Up to 150 °C, water release is observed, which is more intense for the PAI I (COOH) homopolymer. Then, up to 350 °C, significant mass losses are recorded for both homopolymers: up to ~18% for the PAI II (OH) homopolymer and ~15% for the PAI I homopolymer. The DTG curves for the first homopolymer show a broad peak with a maximum at 242 °C and a poorly resolved peak at 300 °C (which corresponds to the overlapping effects of solvent and water release accompanying the cyclization process). For the PAI I (COOH) homopolymer, a broad bimodal peak is also observed on the DTG curve at temperatures of 228 and 326 °C. This peak is related to a sequential release of free and bound NMP solvent at the corresponding temperatures. The latter statement is supported by the TGA and DTG data for PAI I (COOH) homopolymer annealed at 300 °C: there is no peak associated with the release of free solvent on the DTG curve, and a shift of the maximum corresponding to the release of bound solvent to the high-temperature region is observed (Figure 5b).

**Figure 6:**
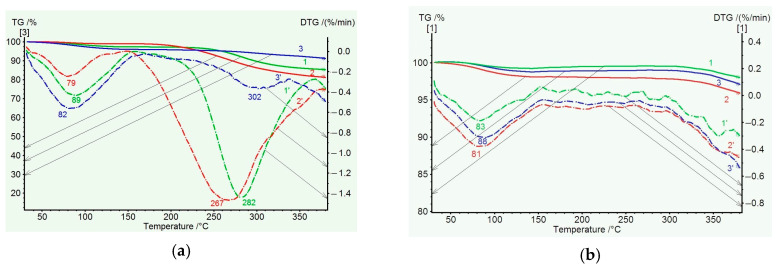
TGA (solid) and DTG (dotted) curves of coPAI samples heated at 150 °C (**a**) and 300 °C (**b**): 1, 1′—coPAI V, 2, 2′—PAI IV, 3, 3′—coPAI III.

Figure 6 illustrates the TGA results obtained for the coPAI samples with different percentages of the hydroxyl-containing fragment DADHyDPhM. In the temperature region up to 150 °C, adsorbed water is released; its relative amount can be determined from the DSC data. In the temperature region up to 350 °C, the mass losses are related to the release of residual solvent and, to a greater extent, to cyclization. In the DTG curve (Figure 6a), the lowest mass loss in this temperature region is recorded for coPAI III, the copolymer with the minimum content of OH groups. Then, the observed mass loss is minimal for coPAI III and more noticeable for coPAI (IV,V) (the samples with higher contents of OH groups). Note that no mass losses are observed for the PAI II homopolymer in this temperature region. Mass losses are registered for all coPAI samples and the PAI I homopolymer.

After heating coPAI at 300 °C, no solvent release is detected. At 320 °C, a minor mass loss is observed due to the release of water during cyclization (Figure 6b). It is most clearly observed for the coPAI V sample, which contains the highest number of fragments bearing OH groups.

**Figure 7:**
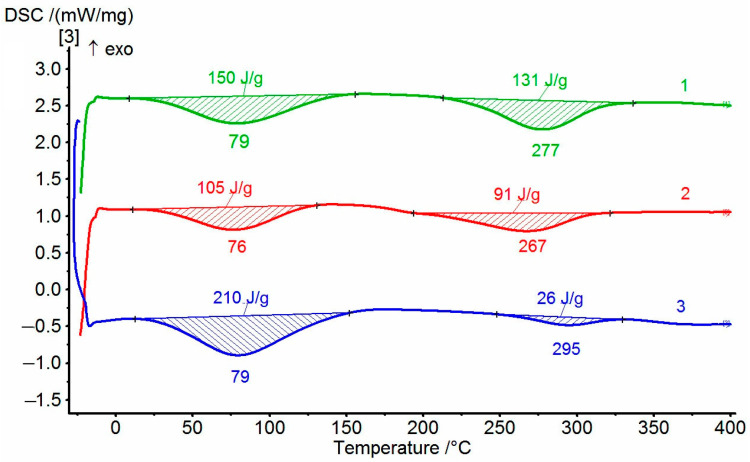
DSC of cope samples after heating at 150 °C: 1—coPAI V, 2—PAI IV, 3—coPAI III.

The dehydrocyclization process was investigated in more detail by DSC. Since this process can have significant energy consumption, the compositions and homopolymer samples were tested in the order of increasing the amount of non-cyclized fragments. Typical thermograms for the coPAI samples treated at different temperatures are shown in Figure 7. During the first scan, the DSC curves show two endotherms reflecting the release of adsorbed water and, at higher temperatures, the completion of the process of cyclization of hydroxyl-containing fragments. For coPAI, the high-temperature endotherm may present the superposition of two processes: the release of residual solvent and cyclization [13]. In this case, the endotherm has a bimodal character and is extended along the temperature axis (the temperature of N-methylpyrrolidone evaporation is 202 °C). In our case, the beginning of the second endotherm is shifted toward higher temperatures. No bimodal endotherms are observed, which may be due either to an increase in the temperature of release of residual solvent or the absence of free solvent. The value of enthalpy of the residual dehydrocyclization process decreases when the sample pretreatment temperature increases (from 100 to 200 °C), and the amount of hydroxyl-containing fragments in coPAI decreases. The results of the studies are summarized in Table 2. An anomalous change in the values of the second endotherm (depending on the content of hydroxyl groups) for coPAI samples after heat treatment at 250 °C draws attention. After heating at 300 °C, no endotherm of residual cyclization is present. The obtained results can be explained by the influence of an acidic environment (the presence of –COOH groups), leading to a significant decrease in the cyclization temperature [28,29].

The analysis of the enthalpy values of the second endotherm of the coPAI samples heated to 150 °C (this temperature is the final one in the process of obtaining film membranes) also showed an abnormally low enthalpy value for coPAI III films (Table 2), which may be due to a lack of hydroxyl groups participating in cyclization. This fact can be explained by the fact that more active carboxyl groups in a complex with the solvent formed intermolecular bonds, which led to a sharp decrease in free hydroxyls, which affects the transport properties of the coPAI films. Note that heating coPAI III to 200 °C led to an increase in the number of hydroxyls due to the removal of the solvent and, accordingly, an increase in the enthalpy value of the second endotherm.

### 3.4. Studies of Intermolecular Interactions

***IR spectroscopy*** was used to confirm the formation of ester bonds between coPAI molecules.

The formation of ester bonds between carboxyl and hydroxyl groups of neighboring macromolecules has been suggested earlier. Since the main bands in the samples overlap, we used difference spectra to confirm the formation of the ester bond. The use of difference spectra obtained by the direct subtraction method is possible in this case, since the “Pike” attachment we use guarantees the same degree of pressing, and, therefore, the same depth of penetration of IR radiation into the sample.

**Figure 8:**
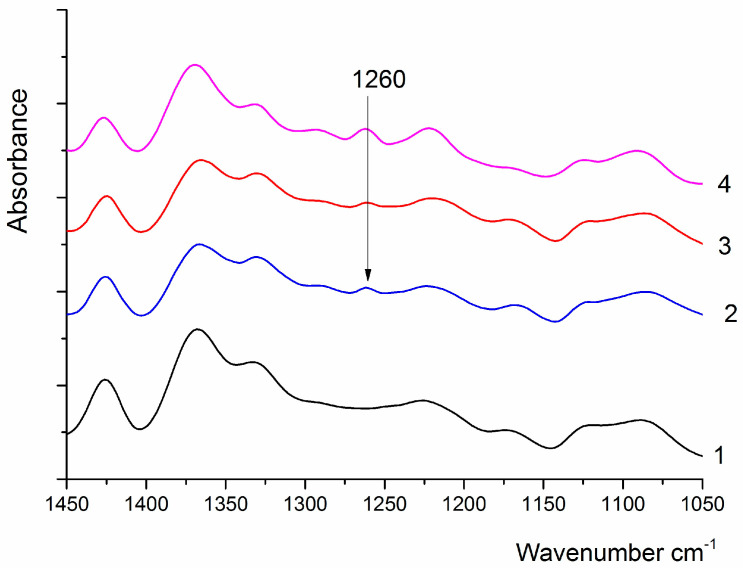
IR spectrum of PAI I (1), difference spectra of coPAI III and PAI II (2), coPAI IV and PAI II (3), coPAI V and PAI II (4).

To confirm this hypothesis, the PAI II spectrum was subtracted from the spectra of coPAI III, coPAI IV, and coPAI V samples, and the difference spectra were compared with the PAI I spectrum (Figure 8). All compared samples underwent the same treatment. In all the difference spectra, the 1260 cm^−1^ band characteristic of C–O valence vibrations in aromatic esters appears. Unfortunately, other characteristic bands (in the region of 1730–1715 cm^−1^ and 1150–1100 cm^−1^) cannot be analyzed in this case because they overlap with the intense bands present in the spectrum.

**Figure 9:**
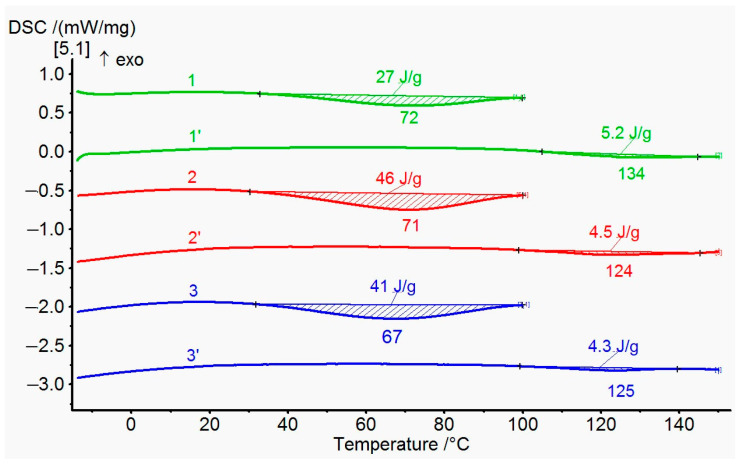
DSC data first and second scan for coPAI: 1, 1′—coPAI V, 2, 2′—coPAI IV, 3, 3′—coPAI III.

An attempt was made to confirm the formation of an ester bond in coPAI III–V by DSC. The results are presented in Figure 9. All the coPAI samples investigated in this experiment were heated according to the following regime: 50 °C (24 h), 80 °C (1 h), and 100 °C (1 h), i.e., below the temperature of formation of ester bond [30]. Then, the coPAI samples were placed in a calorimetric cell, heated to a temperature of 100 °C at a rate of 10 deg/min and kept at this temperature for 5 min to remove adsorbed water. After cooling down to room temperature, the samples were again heated to 150 °C. The thermograms shown in the figure below contain endotherms with a maximum of 123–134 °C. It can be assumed that the observed thermal effects are related to the formation of ester bonds. This effect is most pronounced in the case of coPAI V, which correlates with the light scattering data: the maximum size of associates appeared due to the formation of intermolecular bonds is observed exactly for the copolymer with this ratio between carboxyl and hydroxyl groups (see Section 3.5 “Molecular weights and hydrodynamic characteristics”).

### 3.5. Molecular Weights and Hydrodynamic Characteristics

Table 3 lists the values of molecular weights and hydrodynamic characteristics of PAI (I,II) and coPAI (III–V): weight-average molecular weights *M*_w_, hydrodynamic radii *R*_h-D_, second virial coefficients *A*_2_, characteristic viscosities [*η*] and refractive index increments *dn*/*dc* measured in solutions in NMP and the mixture of solvents (NMP + methanol) at 21 °C.

All polymers have relatively high molecular weights ranging from 42 to 118 kDa.

**Figure 10:**
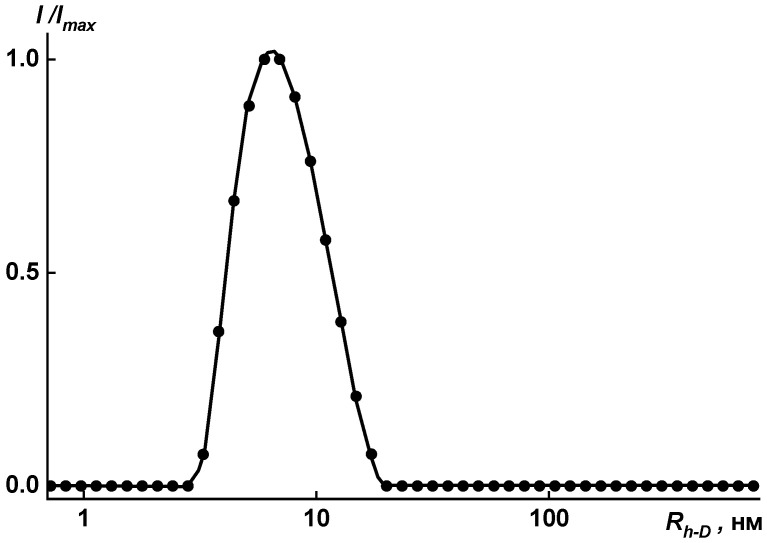
Distribution of hydrodynamic radii for solution of coPAI V (COOH:OH = 30:70) at *c* = 0.0088 g·cm^−3^ in [NMP + methanol]. *I*_max_ is the maximum intensity of scattered light for this concentration.

**Figure 11:**
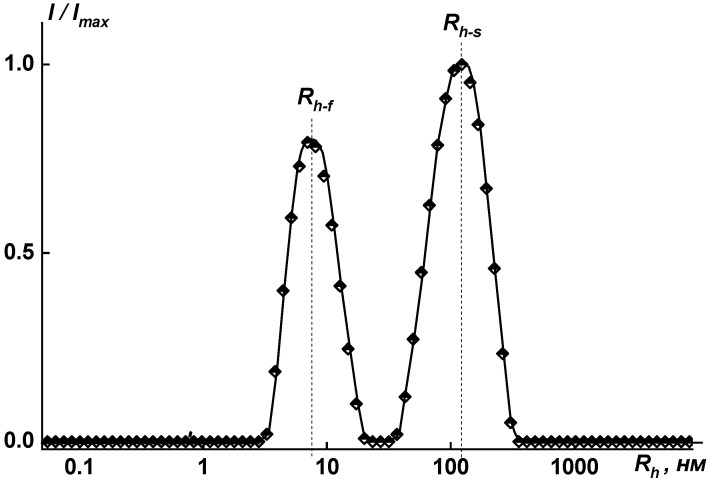
Distribution of hydrodynamic radii for solution of coPAI V (COOH:OH = 30:70) at *c* = 0.0091 g·cm^−3^ in NMP: *R*_h-f_ = 7.5 nm; *R*_h-s_ = 133.5 nm. *I*_max_ is the maximum intensity of scattered light for this concentration.

The size distributions of polymer particles are rather narrow, as is seen in Figure 10 and Figure 11. The unimodal distribution was obtained by dynamic light scattering for the investigated solutions in the mixture of solvents (NMP + 3 drops of methanol) in the concentration range *c* = 0.0010 ÷ 0.0100 g·cm^−3^.

The content of supramolecular structures observed in NMP solutions was less than 0.15 wt.%; therefore, the weight average molecular weight *M*_w_ was calculated from the contribution of individual macromolecules. When pure NMP was used as a solvent, supramolecular structures approximately 100 nm in size (90, 115, and 140 nm) were formed in amounts not exceeding 0.15 wt.%. The largest aggregates are formed in the case of sample coPAI V, in which the ratio of functional groups is COOH to OH = 30 to 70 wt.% (Figure 11). This effect can be explained by high efficiency of intermolecular interactions between chains containing carboxyl functional groups. The higher the amount of carboxyl groups in copolymers, the smaller the size of aggregates.

The values of hydrodynamic radius *R*_h-D_(*c*) did not depend on concentration. Therefore, the concentration-averaged value of *R*_h-D_(*c*) was taken as the hydrodynamic radius *R*_h-D_ for the molecules of the investigated polymer. No asymmetry of light scattering was observed, thus *M*_w_ of the polymer was estimated by the Debye method [31,32].

The obtained positive values of the second virial coefficient *A*_2_ indicate that the thermodynamic quality of the solvents at 21 °C is good. The dependence of the characteristic viscosity on the structure of the amine component is observed. The more fragments with carboxyl groups in the structure of the polymer macromolecule, the higher the value of the characteristic viscosity. This is explained by the higher reactivity of DABA in comparison with DADHyDPhM. The values of characteristic viscosity (*η* determined in the mixture (NMP + 3 drops of methanol) differ slightly from those measured in pure NMP, and their values correspond to the hydrodynamic radii of the macromolecules.

### 3.6. Contact Angles and Surface Free Energy

Investigation of wetting is a convenient method to study the surface properties of solid materials and their changes resulting from surface modification. When the surface layers of films are concerned, competitive intra- and intermolecular interactions in polymers that contain carboxyl and/or hydroxyl functional groups in the repeating units can be considered as a modification; this modification changes the level of film hydrophilicity, wettability, and surface energy (the factors affecting permeability under conditions of pervaporation process).

The data obtained during measurements of water and glycerol contact angles of PAI and coPAI surfaces are presented in Table 4. More comprehensive information about the wetting phenomenon is provided by the knowledge of polar and dispersive components of the surface free energy (SFE) values. The surface energy of the investigated polymer films was calculated, and the results are also presented in Table 4.

Contact angle serves as a quantitative characteristic of the wetting ability of a liquid. The *θ* value characterizes the degree of affinity (or philicity) of the contacting phases. Solids forming an angle *θ* < 90° with water are called hydrophilic, while those with *θ* > 90° are called hydrophobic.

According to Table 4, all samples form contact angles *θ* < 90° with water and glycerol, which indicates their hydrophilicity in the case of water and lyophilicity in the case of glycerol. With the decrease in the amount of carboxyl groups in the series of polymers PAI I, PAI IV, and PAI II, the water contact angle of films naturally increases, and the total surface free energy decreases. However, the change in the dispersive and polar components of the surface free energy in this series of polymers has a complex character. The properties of the copolymer with a lower dispersive component of surface free energy and a higher polar component (compared to homopolymers) attract attention.

Based on the values obtained, the dispersive component of SFE for PAI I and PAI II is significantly larger than the polar component for each of the samples studied. The interactions with the other phase will be determined mainly by van der Waals forces or dispersion forces, i.e., intermolecular interactions. The surfaces of the coPAI IV sample films tend to interact with liquids by means of hydrogen bonding; the bonds are formed by the donor–acceptor mechanism since the polar component of surface free energy predominates over the dispersive component (which, in turn, is due to the presence of polar functional fragments: hydroxyl and carboxyl groups).

**Figure 12:**
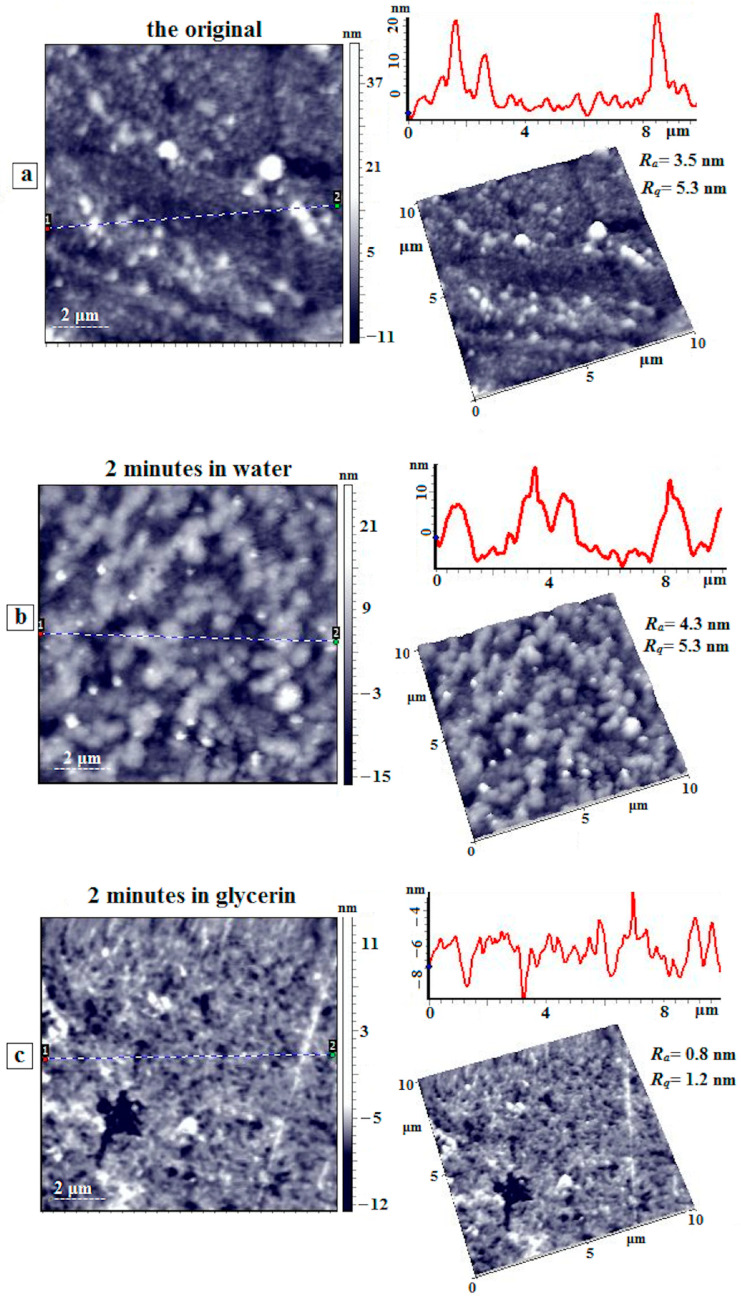
AFM height images, linear profiles (from dot 1 to dot 2), and 3D images of the surface of the coPAI IV (COOH:OH = 50:50) film: (**a**) the initial film, (**b**) the film after exposure to water for 2 min, and (**c**) the film after exposure to glycerol for 2 min.3.7. Surface Morphology of PAI I Films from Dilute Solutions.

Figure 12 presents AFM images of the surface of the initial coPAI IV (COOH:OH = 50:50) film, as well as the film after exposure to water and glycerol for 2 min. On the surface of the initial film (Figure 12a), orientation is clearly observed, which is related to the sample preparation technique (solution casting). Both individual domains and their aggregates located along the orientation axis are visible. After exposure to water (Figure 12b), the morphology of this sample changes significantly. The domains are organized into associates inside the polymer “coat”; the height of these associates above the matrix surface does not exceed 15–20 nm. Similarly to the previous sample, large aggregates are seen with heights reaching 40 nm. Orientation is still observed on the surface, but it is not as pronounced as that in the initial film. At the same time, the values of the arithmetic mean (R_a_) and root mean square (R_q_) deviation of the profile practically do not change and are equal to 3.5 nm and 5.3 nm for the original film and 4.3 nm and 5.3 nm for the film exposed to water.

When the coPAI IV film is exposed to glycerol (Figure 12c), significant surface smoothing occurs. The values of profile deviations are R_a_ = 0.8 nm and R_q_ = 1.2 nm. The surface becomes loose, and numerous caverns with diameters of 100–200 nm and large micron-sized caverns are observed on the surface, where the depth of some of them reaches 15 nm. No pronounced orientation is observed, unlike the initial sample.

To summarize, contact with polar liquids leads to changes in the structural organization of polymer films near the working surface of the membrane. However, no increase in the surface roughness of the samples is detected. The observed structural rearrangement occurs as a result of interaction with molecules of polar liquids without the release of any substances from the membrane surface. The obtained data correlate well with the pervaporation results described above. The increase in cyclohexane permeability after the transport of polar penetrants through the membrane may be related to the increase in free volume in the films of polymers containing carboxyl groups. The obtained membranes are generally stable after direct contact with the tested penetrants.

**Figure 13:**
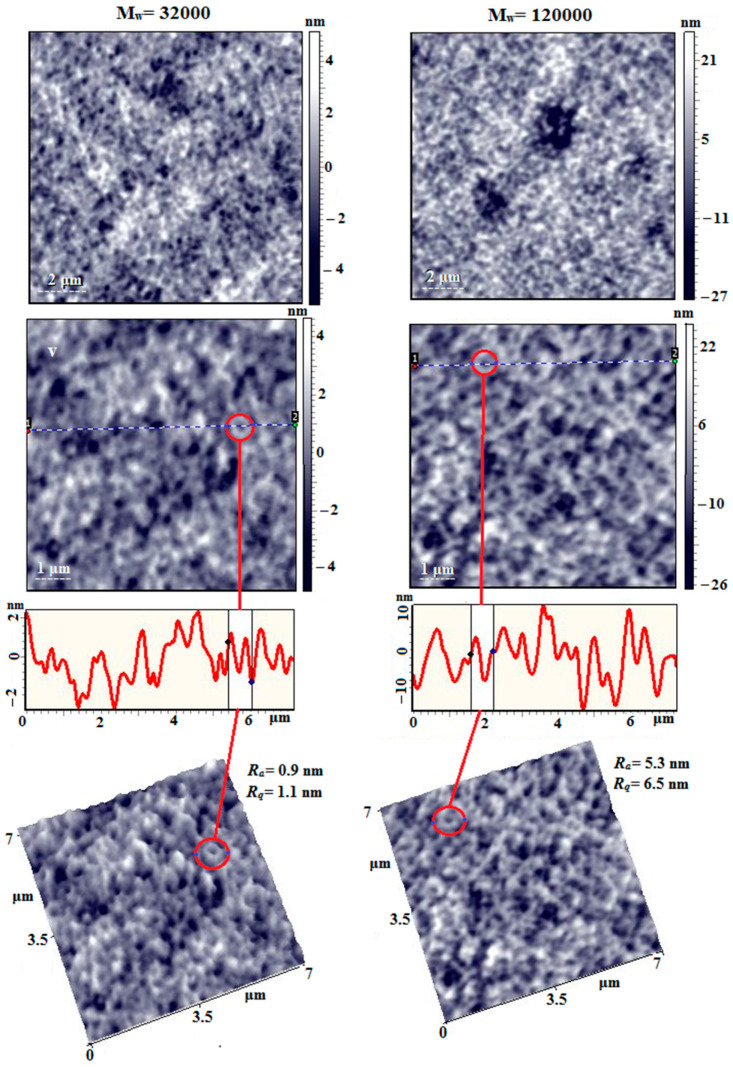
AFM height images, linear profiles (from dot 1 to dot 2), and 3D images of the surface of PAI I films obtained from dilute solutions on the surface of a fresh mica chip.

**Figure 14:**
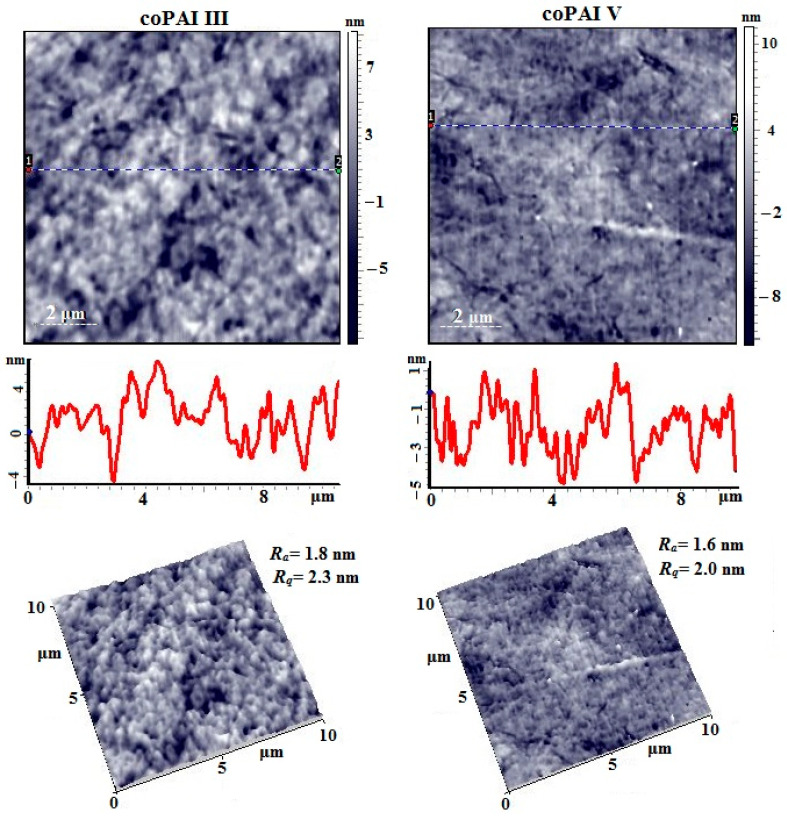
AFM height images, linear profiles (from dot 1 to dot 2), and 3D images of the surface of coPAI III and coPAI V films.

AFM images of the PAI I homopolymer were obtained from dilute solutions (0.5–2.0 g/dL) held for long periods of time. Long-term holding resulted in intermolecular cross-linking and the formation of supramolecular structures, as shown in Figure 13 and Figure 14.

Figure 13 shows AFM images of films obtained from dilute solutions of PAI I homopolymer with different molecular weights. On the surface of the film with Mw = 32,000 Da, a loose, nanoporous, spongy morphology is recorded, with a nanopore diameter of 100 ÷ 250 nm. Also clearly visible are chain aggregates with nodular formations, intertwined with each other, some of which have a closed shape in the form of a torus, the outer diameter of which is 200 ÷ 400 nm.

The surface of the film made of PAI-I with Mw = 120,000 Da is similar in morphology to the previous one; however, the surface roughness values are ~5 times greater and are R_a_ = 0.9 nm and R_q_ = 1.1 nm for the low-molecular PAI I sample and R_a_ = 5.3 nm and R_q_ = 6.5 nm for the high-molecular PAI I sample with a scanning matrix of 7 µm × 7 µm. On the film surface, nodular formations are more clearly visible against the background of chain aggregates. Many formations with a torus geometry are also visible, the outer diameter of which is similar to the previous sample.

Figure 14 shows the AFM images of the surface of coPAI III and coPAI V films. A large number of caverns with a maximum depth of ~9 nm are visible on the surface of the coPAI III film sample. A large number of formations with a torus geometry are observed, tightly adjacent to each other. The surface roughness value is R_a_ = 1.8 nm and R_q_ = 2.3 nm (for a 10 µm × 10 µm scanning matrix). The surface of the coPAI V film is denser and smoother. The caverns are mainly very small, their diameter is 50 ÷ 80 nm, and the depth is no more than 6 nm. The surface roughness value is practically no different from the previous sample and is R_a_ = 1.6 nm and R_q_ = 2.0 nm (for a 10 µm × 10 µm scanning matrix).

Thus, it can be concluded that the morphology of toroidal formations observed for the homopolymer PAI I is also reproduced in coPAI III with the maximum number of fragments containing carboxyl groups, which is determined by the presence of the NMP solvent bound to the carboxyl groups.

## 4. Conclusions

The membranes based on polyamide-imides containing carboxyl and/or hydroxyl groups in the repeating units of macromolecules were synthesized and investigated for the first time. The introduction of fragments with these functional groups into polymer chains makes it possible to influence the realization of intra- and intermolecular interactions and, as a consequence, the number of functionally active carboxyl groups that determine the surface properties of membranes. Although the formation of ester bonds is realized in the process of membrane production at the stage of post-treatment of cast films, this process is less energetically favorable than other transformations with the participation of carboxyl groups occurring in the system. The observed effects that influence the character of the interaction of these polymers with polar penetrants are responsible for high levels of membrane permeability accompanied by high selectivity for mixtures of liquids with significantly different polarities. This is evidenced by high values of the ideal membrane selectivity coefficient in the separation of the methanol/cyclohexane mixture, which, for example, is equal to 92 in the case of coPAI III.

The presented results suggest that to form an effective separation membrane, it is not sufficient to introduce functional groups interacting with a penetrant into a membrane-forming polymer; it is also necessary to enhance their functional activity.

## Figures and Tables

**Table 1:** Pervaporation properties of dense membranes at 40 °C. The order of penetrants during pervaporation: (I) cyclohexane (Cyh) → ethanol (EtOH) → methanol (MeOH) → water (HOH); (II) HOH → MeOH → EtOH → Cyh.

Sample	*l*, µm	P·*l*, µm∙kg·m^−2^∙h^−1^				
		Cyclohexane, 100%	Ethanol, 96%	Methanol, 100%	Water, 100%	The Order of Penetrants
PAI II	52.5	0.074 ± 0.004	1.3 ± 0.1	2.3 ± 0.1	0.50 ± 0.03	→
		0.063 ± 0.003	0.10 ± 0.01	0.84 ± 0.04	0.77 ± 0.04	←
coPAI V	42	0.37 ± 0.02	0.38 ± 0.02	5.2 ± 0.3	5.6 ± 0.3	→
		0.27 ± 0.01	0.25 ± 0.01	2.5 ± 0.1	5.1 ± 0.3	←
coPAI IV	30	0.38 ± 0.02	1.00 ± 0.05	4.8 ± 0.2	6.0 ± 0.3	→
		2.4 ± 0.1	2.4 ± 0.1	7.1 ± 0.4	5.2 ± 0.3	←
coPAI III	25	0.090 ± 0.005	2.1 ± 0.1	8.3 ± 0.4	4.6 ± 0.2	→
		0.20 ± 0.01	1.5 ± 0.1	2.7 ± 0.1	5.9 ± 0.3	←
PAI I	40.5	0.25 ± 0.01	2.4 ± 0.1	5.1 ± 0.3	7.0 ± 0.4	→
		0.34 ± 0.02	0.23 ± 0.01	2.5 ± 0.1	7.9 ± 0.4	←

**Table 2:** DSC data for compositions and homopolymers.

Composition DABA: DADHyDPhM	Temperature of Treatment, °C	T_max_, °C Endotherm 1	ΔH, J/g Endotherm 1	T_max_, °C Endotherm 2	ΔH, J/g Endotherm 2
100:0	150	75.6	139.3	-	-
	200	77.2	221.7	-	-
	250	78.0	213.5	-	-
	300	94.6	145.3	-	-
0:100	300	73.0	40.35	336	20.68
70:30	300	83.8	151.2	-	-
	250	81.0	124.8	296.5	26.79
	200	75.6	105.6	272.4	69.75
	150	79.3	210.1	295.0	25.79
30:70	300	81.3	61.2	-	-
	250	78.2	110.1	310.1	12.87
	200	78.4	63.54	270.8	135.2
	150	78.6	150.3	277.3	131.0
50:50	300	79.9	103.4	-	-
	250	75.7	74.94	293.5	71.01
	200	79.9	206.0	289.8	60.05
	150	76.3	105.0	267.2	90.86

**Table 3:** Molecular weights and hydrodynamic characteristics of PAI (I,II) and coPAI (III–V) in NMP and the NMP-containing mixture (NMP + 3 drops of methanol).

Sample	Solvent	*dn*/*dc*, cm^3^ g^−1^	M_w_, kDa	*R*_h-D_, nm	*A*_2_, cm^3^·mol·g^−2^	[*η*], cm^3^·g^−1^	Density/Buoyancy Factor g/cm^3^
PAI I	NMP	0.1935	41.9	4.8–5.1	5.12 × 10^−4^	57.69	1 − ν_2_ρ = 0.3385 ν_2_ = 0.6406
coPAI III	NMP	0.2045	62.3	5.8 av.	6.85 × 10^−4^	69.06	1 − ν_2_ρ = 0.3323 ν_2_ = 0.6466
	NMP + 3 drops of methanol	0.2177	63.3	5.6 av.	3.38 × 10^−3^	55.2	-
coPAI IV	NMP	0.2059	80.5	6.8 av.	1.14 × 10^−3^	77.3	1 − ν_2_ρ = 0.3301 ν_2_ = 0.6488
	NMP + 3 drops of methanol	0.2136	79.2	6.5 av.	4.13 × 10^−3^	66.6	-
coPAI V	NMP	0.2053	100.1	7.5	1.45 × 10^−3^	87.8	1 − ν_2_ρ = 0.3208 ν_2_ = 0.6578
	NMP + 3 drops of methanol	0.2188	100.9	7.5	4.61 × 10^−3^	75.3	-
PAI II	NMP	0.2100	117.6	8.8–9.5	2.88 × 10^−3^	93.3	1 − ν_2_ρ = 0.2950 ν_2_ = 0.6827
	NMP + 3 drops of methanol	-	-	-	-	87.79	-

**Table 4:** Wettability and energy characteristics of surfaces of the tested samples.

Sample	Contact Angle *θ*, °		Surface Free Energy		
	Water	Glycerol	Dispersion Component, *γ^d^*, mJ/m^2^	Polar Component, *γ^p^*, mJ/m^2^	Total Surface Energy, *γ*, mJ/m^2^
PAI I (m = 1, n = 0)	54.6 ± 1.7	39.8 ± 0.2	32.20	18.41	50.61
coPAI IV (m = n = 0.5)	63.3 ± 0.3	55.5 ± 0.6	19.27	20.06	39.33
PAI II (m = 0, n = 1)	67.8 ± 0.3	57.3 ± 0.2	24.15	14.19	38.34

## Data Availability

Data is contained within the article or Supplementary Materials.

## Supplementary Materials

The following supporting information can be downloaded at: https://www.mdpi.com/article/10.3390/membranes15010023/s1, Figures S1–S5: NMR data ^13^C of PAI I, coPAI IV, PAI II, coPAI III samples.
