# Supplementary material for: Realization of Intermolecular Interactions as a Basis for Controlling Pervaporation Properties of Membranes Made of Aromatic Polyamide-Imides
Source: Membranes (Basel). 2025 Jan 13;15(1):23. doi: 10.3390/membranes15010023 (PMC11766727; doi:10.3390/membranes15010023)
Supplement: Supplementary file 1 [file membranes-15-00023-s001.zip › membranes-3380060-supplementary.pdf]

Supplamentary

Realization of intermolecular interactions as a basis for controlling pervaporation properties of membranes made of aromatic polyamide-imides

Svetlana V. Kononova[1*], Galina N. Gubanova[1*], Galina K. Lebedeva[1], Elena V. Kruchinina[1], Elena N. Vlasova[1], Elena N. Popova[1], Natalya V. Zakharova[1], Milana E. Vylegzhanina[1], Elena A. Novozhilova[2], Ksenia V. Danilova[2]

* Corresponding authors: gubanovagn@yandex.ru

NMR data of PAI's

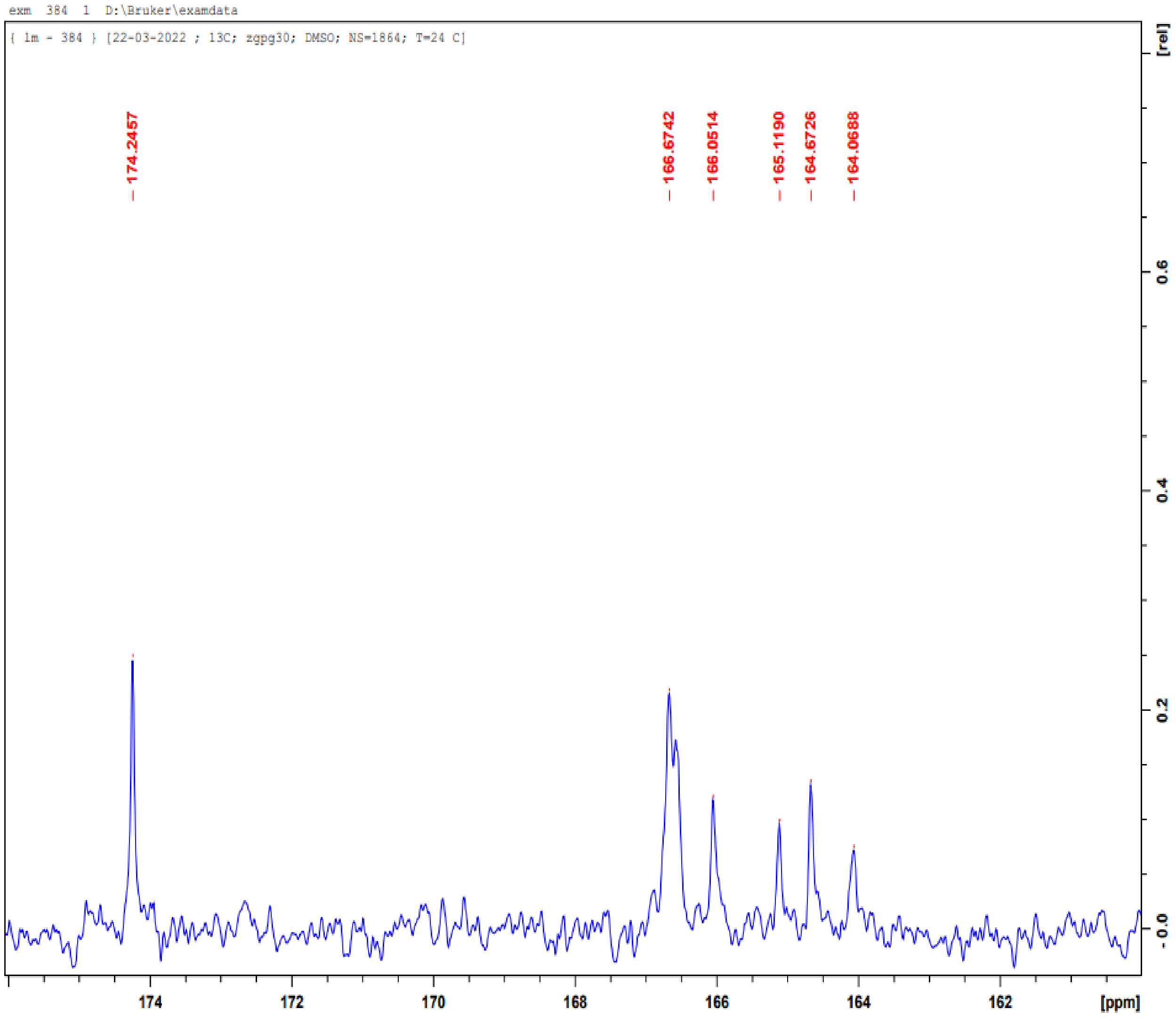

Fig.S1 – NMR data ^{13}C of PAI – I sample

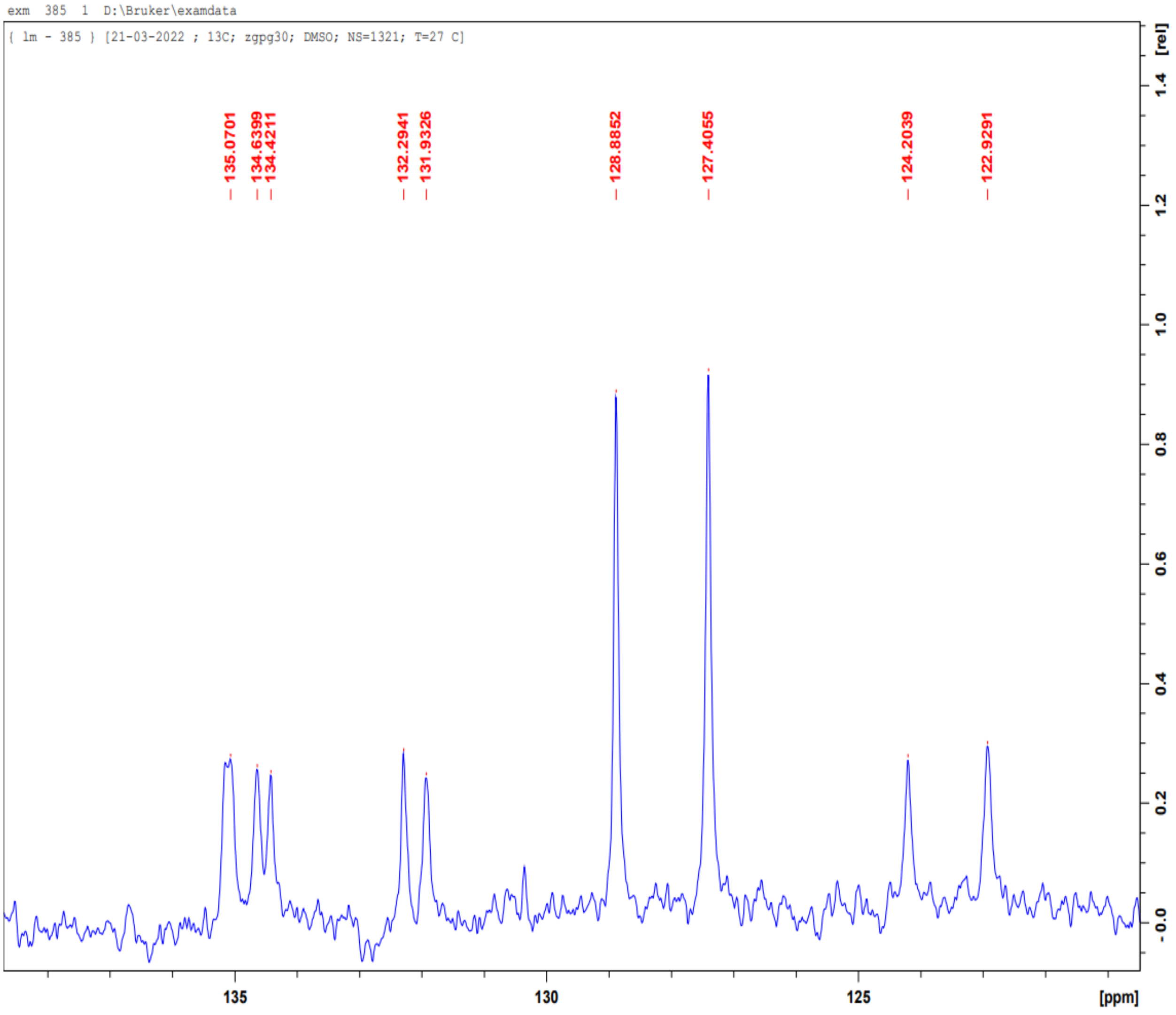

Fig.S2 – NMR data ^{13}C of coPAI- IV sample

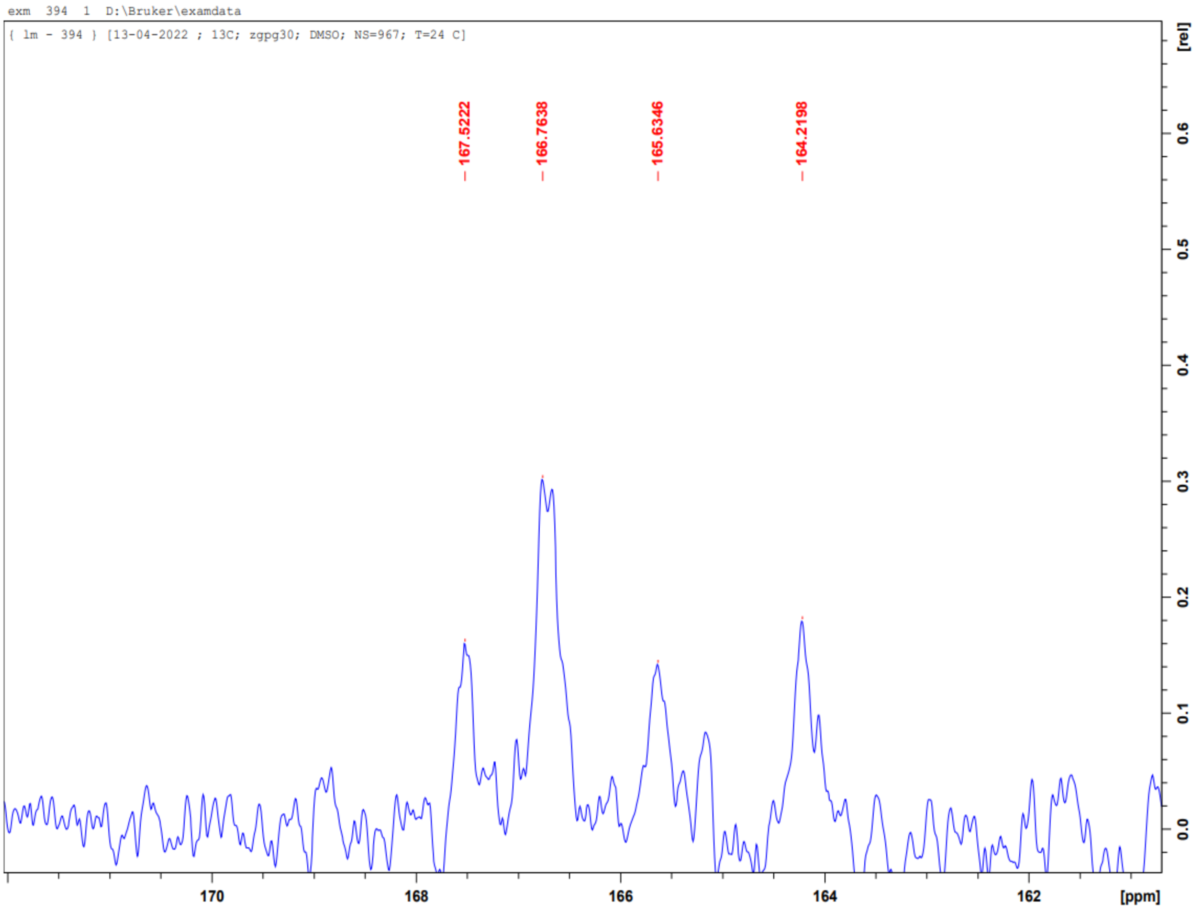

Fig.S3 – NMR data ^{13}C of coPAI- IV sample

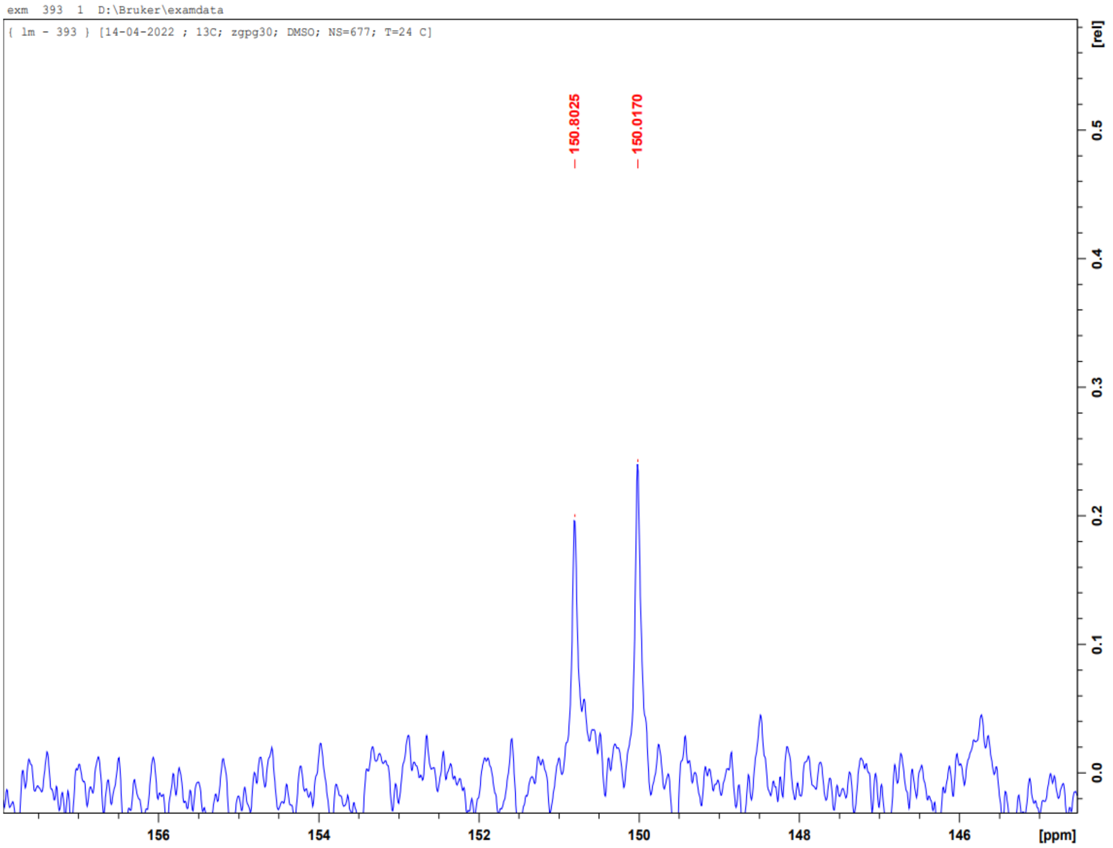

Fig.S4 – NMR data ^{13}C of PAI- II sample

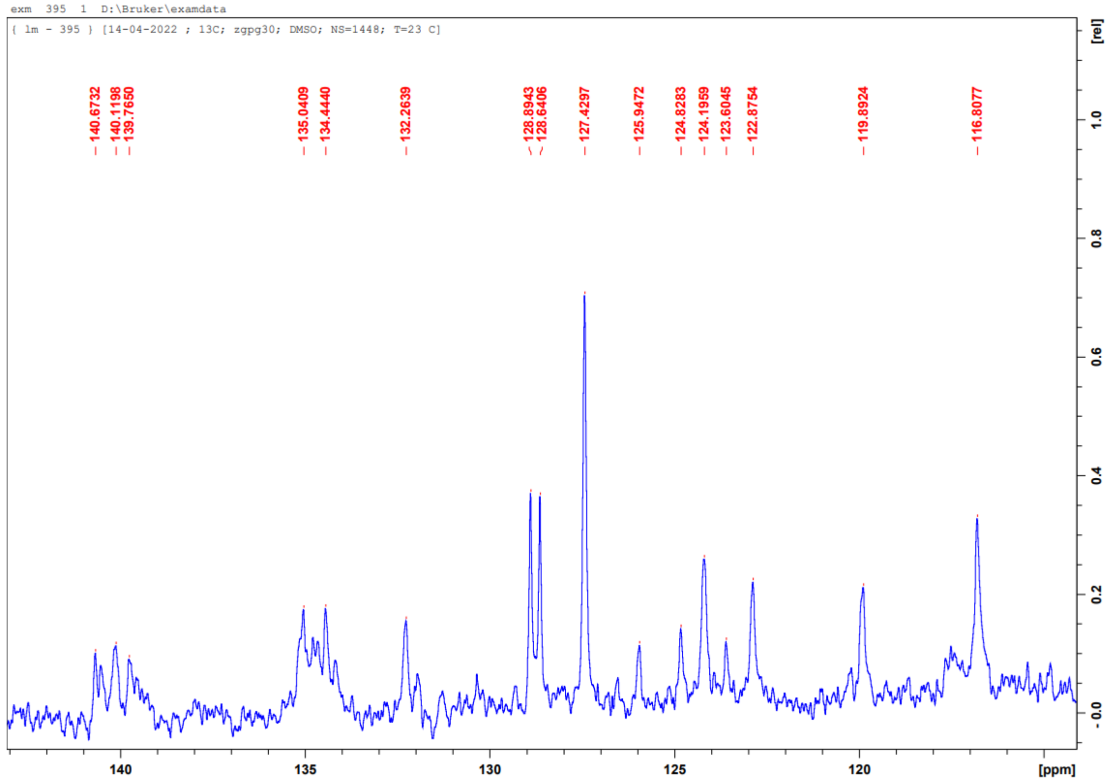

Fig.S5 – NMR data ^{13}C of coPAI- III sample
